# Ultra-Sensitive Strain Sensor Based on Flexible Poly(vinylidene fluoride) Piezoelectric Film

**DOI:** 10.1186/s11671-018-2492-7

**Published:** 2018-03-14

**Authors:** Kai Lu, Wen Huang, Junxiong Guo, Tianxun Gong, Xiongbang Wei, Bing-Wei Lu, Si-Yi Liu, Bin Yu

**Affiliations:** 1 State Key Laboratory of Electronic Thin Films and Integrated Devices, University of Electronic Science and Technology of China, Chengdu, 610054 China; 2 State Key Laboratory of Luminescent Materials and Devices, South China University of Technology, Guangzhou, 510006 China; 3 AML, Department of Engineering Mechanics, Tsinghua University, Beijing, 100084 China; 4 College of Nanoscale Science and Engineering (CNSE), State University of New York, Albany, NY 12203 USA

**Keywords:** Piezoelectricity, PVDF film, Tactile pressure, Flexible sensor

## Abstract

A flexible 4 × 4 sensor array with 16 micro-scale capacitive units has been demonstrated based on flexible piezoelectric poly(vinylidene fluoride) (PVDF) film. The piezoelectricity and surface morphology of the PVDF were examined by optical imaging and piezoresponse force microscopy (PFM). The PFM shows phase contrast, indicating clear interface between the PVDF and electrode. The electro-mechanical properties show that the sensor exhibits excellent output response and an ultra-high signal-to-noise ratio. The output voltage and the applied pressure possess linear relationship with a slope of 12 mV/kPa. The hold-and-release output characteristics recover in less than 2.5 μs, demonstrating outstanding electro-mechanical response. Additionally, signal interference between the adjacent arrays has been investigated via theoretical simulation. The results show the interference reduces with decreasing pressure at a rate of 0.028 mV/kPa, highly scalable with electrode size and becoming insignificant for pressure level under 178 kPa.

## Background

Poly(vinylidene fluoride) (PVDF) is a chemically stable piezoelectric polymer material that has many applications in different fields for its pyroelectric, piezoelectric, and ferroelectric properties [1, 2]. Especially, owing to the outstanding mechanical properties (the Young’s modulus 2500 MPa and strength at break point ~ 50 MPa), the pressure sensor based on PVDF shows a good mechanical property such as flexibility and antifatigue [3, 4]. Compared with the commonly used pressure sensors based on ferroelectric PZT family materials, the PVDF-based pressure sensor is nontoxic and biocompatible [5, 6]. Most importantly, the PVDF-based sensor was more soft and tough than PZT-based sensor due to the high flexibility coefficient of PVDF film, which could be made the required shapes for complex strain sensing [7, 8]. Accordingly, the PVDF-based pressure sensor is thought to be one of the potential flexible bio-sensor for pressure characterization in the rapid development of bio-medical field [9, 10]. Sharma et al. designed a pressure sensor for smart catheter with PVDF film; it could be integrated onto a catheter for real-time pressure measurement [11]. Bark et al. developed a pulse wave sensor system to non-intrusively measure heart pulse wave signals from driver’s palms based on PVDF; results show that the sensor system can provide clear pulse wave signals for heart rate variability analysis, which can be used to detect driver’s vigilant state to avoid traffic accidents [12]. Lee et al. fabricated a sensor with PVDF and ZnO nanostructures and it could detect the changes in pressure and temperatures for artificial skin [13]. The sensor, however, only detects pressure at a single point with large dimension.

Real-world applications, such as patched biosensor for detecting the human body pressure, demand multipoint sensing, structurally flexibility, and ultra-high sensitivity [14–16]. In this reported work, a 4 × 4 flexible sensor array based on piezoelectric PVDF film is demonstrated, showing ultra-high sensitivity of 12 mV/kPa and fast output response of 2.5 μs. The magnitude and spatial distribution of the pressure applied on a human finger are characterized.

## Design and Experimental

### Design and Fabrication of the Sensor Array

**Fig. 1:**
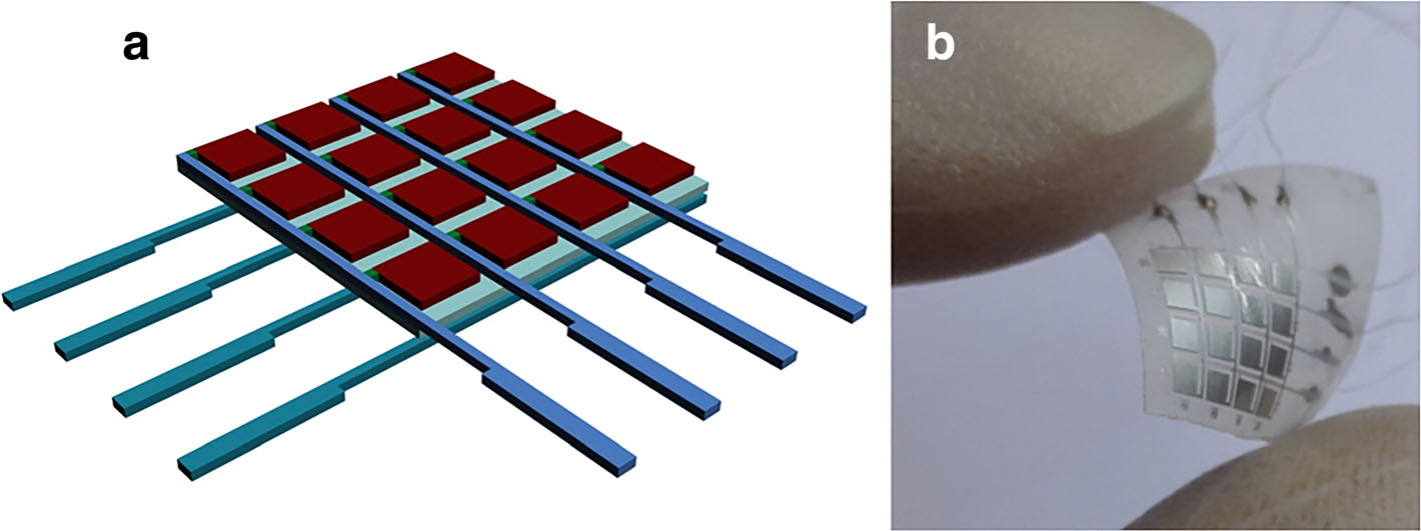
**a** Schematic diagram of the sensor array. **b** Physical picture of the ultimate device

The proposed sensor array has a sandwich structure based on a PVDF thin film with the thickness of about 50 μm (Jinzhou Kexin Inc., China). The aluminum electrode arrays with the thickness of 20 μm were covered on both sides of the PVDF film. Figure 1a shows a schematic design of the sensor. The sensor has 16 micro-capacitor units; every 4 units share one connecting wire to minimize the amount of the electrode wires.

To fabricate the sensor array, a slide glass covered with polydimethylsiloxane (PDMS) was prepared as a stiff substrate. The PVDF thin film covered by Al on both sides was loaded on the substrate. Then, the photoresist was spin-coated on the surface of the film with a speed of 3000 rpm for 40 s. After photolithography and wet etching of Al by a mask aligner system (ABM, Inc., USA), the 16 capacitor units with 4 × 4 square structure were prepared. After that, the flexible sensor on the PDMS substrate was picked up from the slide glass. The electrodes of each capacitor were connected with the conductive wires through silver glue. In order to obtain good bio-compatibility, the sensor was packaged by being covered with PDMS on the top and heated for 12 h at 60 °C. Figure 1b displays a photograph of the bent pressure sensor, illuminating that the sensor is flexible.

### Piezoelectric Property of the Sensor Array Based on the PVDF Film

Piezoresponse force microscopy (PFM) study (Seiko, Inc., Japan) was carried out to characterize the surface morphology and piezoelectric properties of the PVDF film of the proposed sensor under an AC bias voltage of 2 V with a scanning area size of 2 × 2 μm^2^.

### Calibration for the Sensor Array

To calibrate the sensor, various pressures were applied on the proposed sensor in an electro-mechanical experimental platform connecting to a data acquisition (DAQ-USB6008) equipment from National Instruments. The data acquisition with four differential analog signals was set with differential model. The output voltage signal from the proposed sensor was obtained by changing the connection between sensor array and the DAQ.

## Results and Discussion

**Fig. 2:**
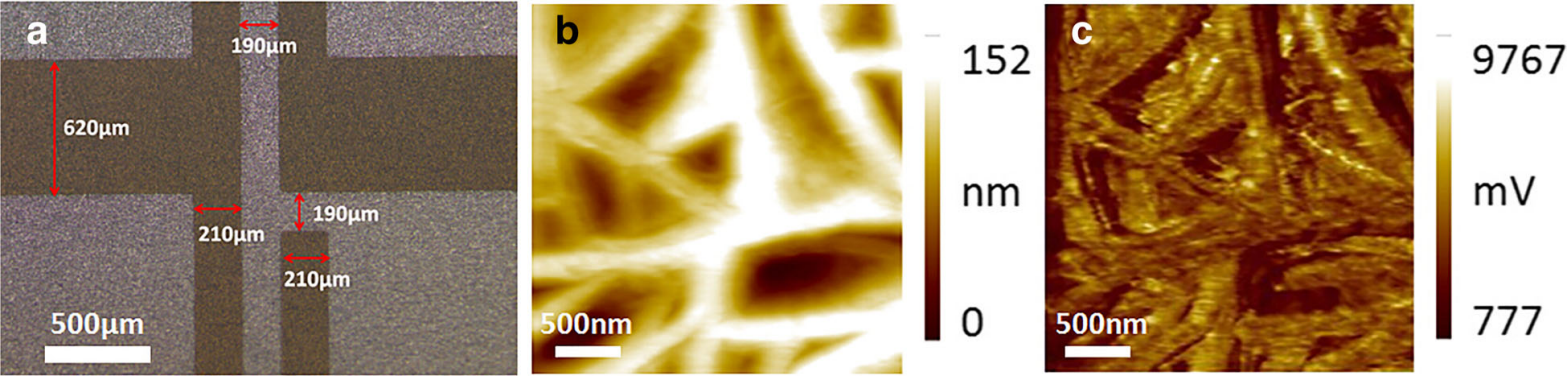
**a** Surface morphology of the proposed sensor after etching technology. **b** Surface morphology and **c** phase PFM images of PVDF film of the sensor

Figure 2a shows the surface morphology of the sensor after etching of Al, checked by an optical microscope. The fairly bright and dark contrast suggests a clear interface between PVDF and the etched Al electrodes. Figure 2b, c shows the surface morphology and phase signal of the PVDF film of the pressure sensor. It is indicated that the surface of PVDF is smooth with a tissue structure. The phase image of PFM measure in Fig. 2c shows a strong response of the piezoelectric domain which is consistent with the surface structure seen in Fig. 2b. These results suggest that the as-prepared sensor based on the PVDF film exhibits a good piezoelectricity.

**Fig. 3:**
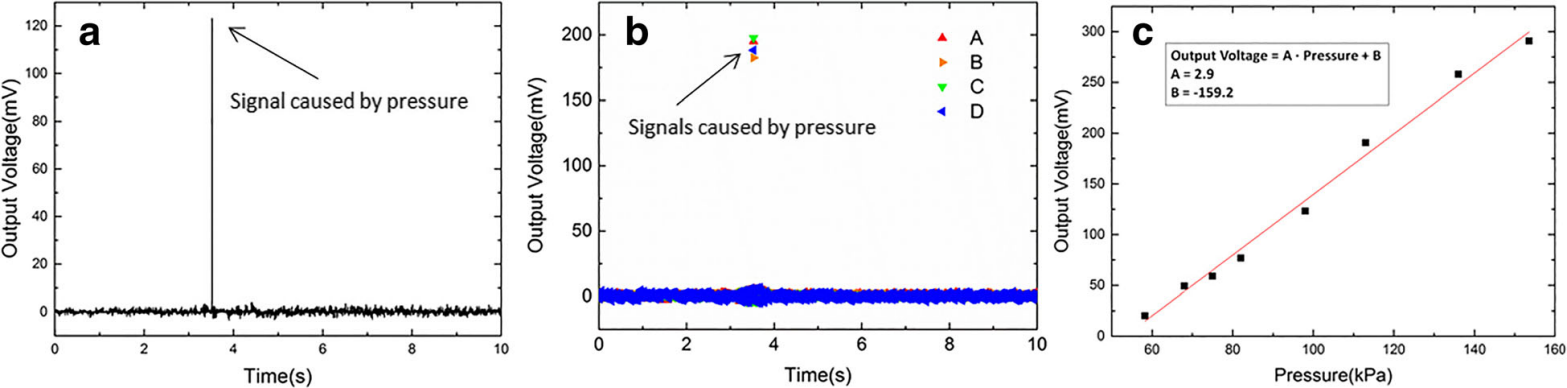
Filtered output voltages for **a** an electrode square and **b** four electrode squares of the sensor array. **c** Liner calibration curve fit of the proposed sensor

A typical result of the output signal is shown in Fig. 3a when a constant pressure of 98.1 kPa was applied on one of the squared electrode of the sensor [17]. The *x*-axis and *y*-axis show the time and the output voltage of the squared electrode of the sensor, respectively. The output voltage was converted from charge (Q) generated by the PVDF film of the sensor. Based on the piezoelectricity equation (where *d*_33_ is a piezoelectric constant when the direction of polarization is the same with the direction of electric field and F_Z_ means pressure is applied on the *z*-direction with the same direction of *d*_33_), a relation between output voltage and pressure could be established. The raw data were obtained by applying a band block of 49–51 Hz. The arrow line of this figure indicates the signals of about 123.1 mV which was generated by the pressure applied on the sensor. The output voltage of the sensor by the pressure is shown clearly in the signal with low noise and high signal-to-noise ratio. In order to confirm the synchronal property of the sensor array, an equal pressure of 113.2 kPa was applied on four units of the sensor, simultaneously. The output voltage signals induced by the pressure were showed in Fig. 3b. The nearly same output value of about 190 mV was obtained from the four units of the sensor at the same time, which suggests that the sensor array exhibited a high stability and synchronal property by applying multipoint pressure. For calibrating the sensor array, different pressures in the range of 60–150 kPa were applied on the sensor array; the output voltage vs. the applied pressure were obtained and plotted as the calibration curve shown in Fig. 3c, which exhibits a linear relationship. The slope of the linear curve is about 2.9 mV/kPa, and there is an offset of − 159.2 mV in the calibration curve.

**Fig. 4:**
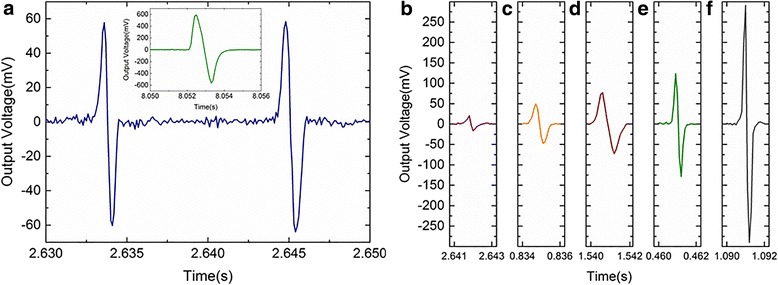
The hold-and-release output response from the pressures of **a** 75.1 kPa, **b** 58.2 kPa, **c** 67.8 kPa, **d** 81.9 kPa, **e** 98.1 kPa, and **f** 153.6 kPa; the inset shows the hold-and-release output response obtained from bare PVDF film

The hold-and-release output response of one squared electrode of the sensor was obtained by applying an impulse pressure with various frequencies. The plotted curve in Fig. 4a shows the typical response of the sensor by applying the impulse pressure of about 75.1 kPa with a frequency of 90 Hz. The positive output voltage corresponds to the compression of the electrode square of the sensor array, and the negative output voltage corresponds to the relaxation. As seen in the inset of Fig. 4a, the similar hold-and-release output response has also been observed in the bare piezoelectric PVDF film [18]. The response time of output voltage of the sensor is less than 2 ms, which suggests the sensor exhibits a good electro-mechanical response property. The impulse pressures within the range of 60–150 kPa were applied on the sensor array. The hold-and-release output response curves were shown in Fig. 4b. The sensor shows a stable characteristic of electro-mechanical response with the response time of about 2 ms under different pressures, and the output voltages of the sensor under different pressures are consistent with the linear calibration curve obtained above.

**Fig. 5:**
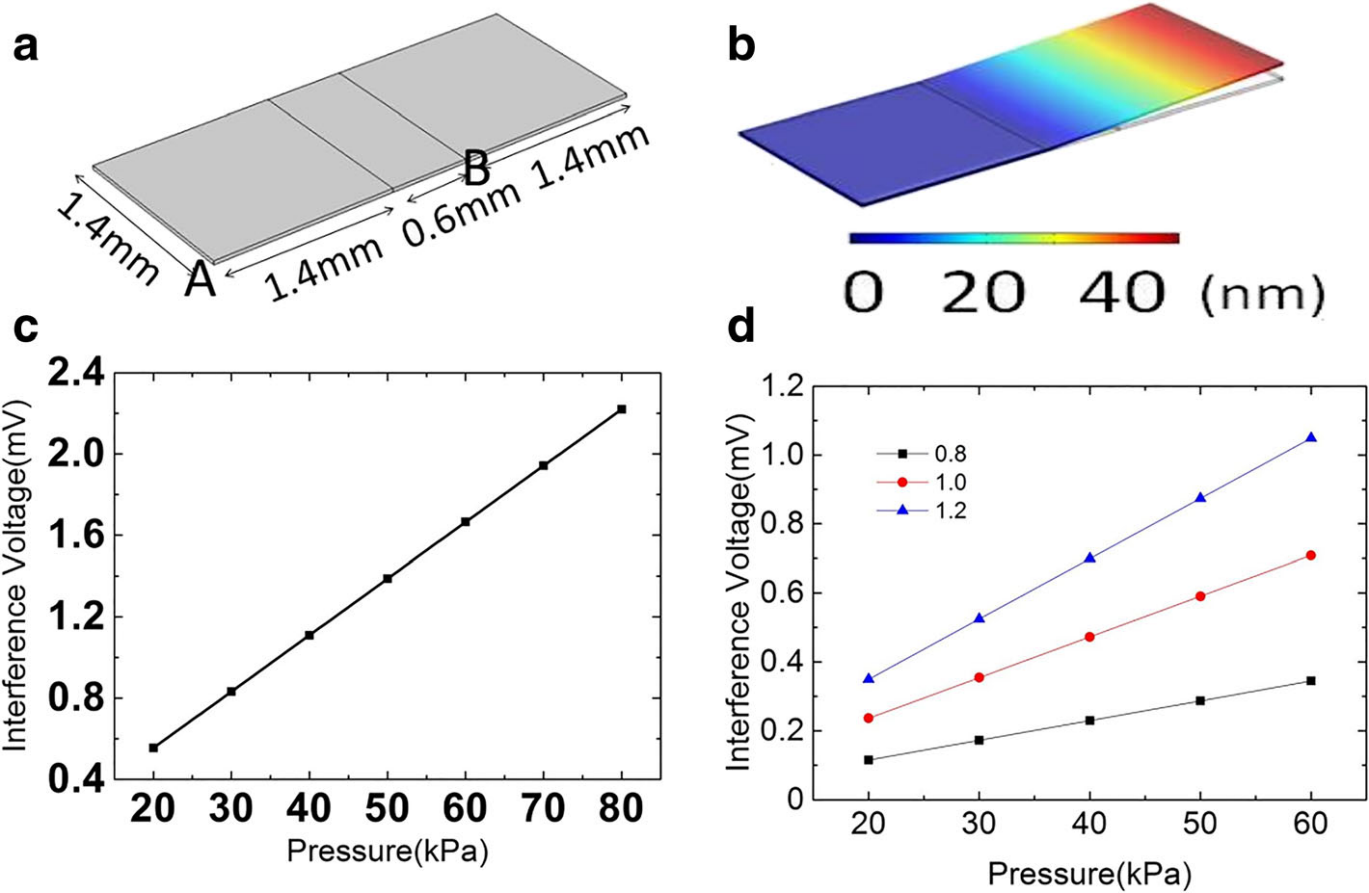
**a** Physical dimensions used for theoretical simulation. **b** Displacement and **c** liner curve-fitting between interference voltage and applied pressure with an array size of 1.4 mm. **d** Obtained results using array sizes of 0.8, 1.0, and 1.2 mm, respectively

Next, application of pressure on selective point is studied. Signal interference is shown between the adjacent arrays, when pressure was applied on the electrode of one of the arrays. The simulation of signal interference was conducted via COMSOL Multiphysics on arrays. Each electrode area is 1.4 mm^2^. The geometry of the structure is shown in Fig. 5a. The additional strain, when pressure was applied on electrode A, is seen in Fig. 5b, indicating the strain increases with the distance away from electrode A. The interference in potential difference with a pressure level of 20~80 kPa was studied, shown in Fig. 5c. The potential difference and pressure exhibit a linear relationship with a slope of 0.028 mV/kPa and an intercept of 5 × 10^−4^ mV, implying very-low-level interference. A pressure under 178 kPa would generate signal interference less than 5 mV which is negligible [16, 17]. In addition, the dependence of interference on array electrode size has been investigated. Figure 5d shows the result with electrode sizes of 1.2, 1.0, and 0.8 mm^2^. It shows that a linear relationship between interference potential difference and pressure (in the range of 20~60 kPa) can be still observed in the smallest electrode. The fitting slopes for interface voltage are 0.01748, 0.01181, and 0.00574 mV/kPa, respectively, for the three structures with the noted observation of reduced interference potential in smaller electrode size.

**Fig. 6:**
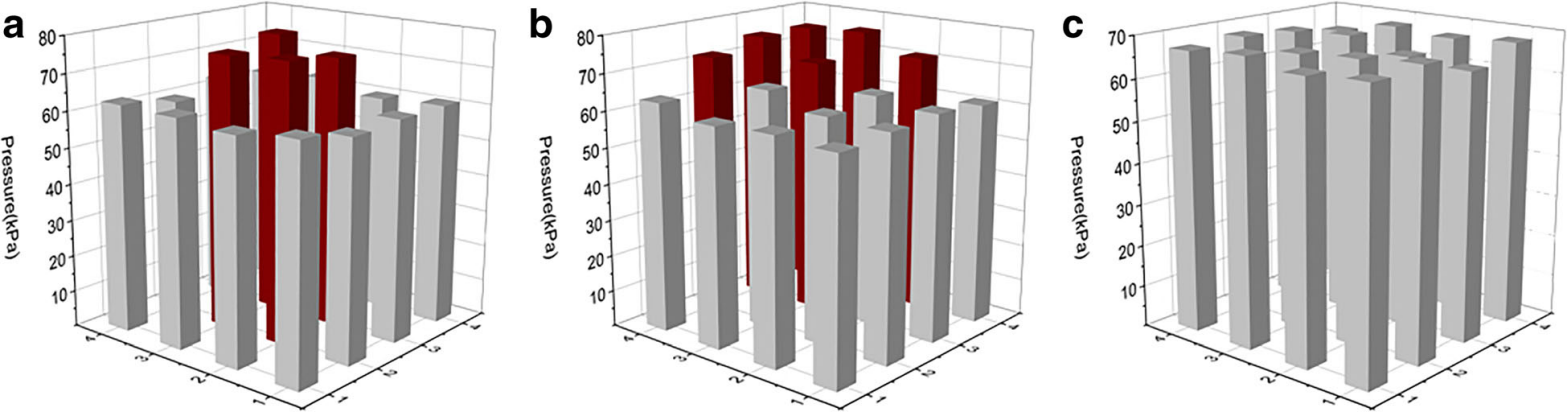
The pressure state and distribution of the thumb finger movement characterized by the proposed sensor: **a** the shiatsu, **b** the kneading, and **c** the rub

For a simple practical application, the sensor was applied to measure the pressure state and distribution of the finger of human hand. As we all have known, the complex finger movement consists of some basic skills, such as shiatsu, kneading, rub, friction, and so on [19]. In our experiments, three most commonly used movements including shiatsu, kneading, and rub were selected to test the pressure state and distribution of the finger. Figure 6 shows a snap of the pressure distribution of the thumb finger characterized by the sensor during the three movements of the finger, respectively. In Fig. 6a, it could be clearly seen that the pressure of 76 kPa was focused in the center of the thumb finger during the shiatsu movement, which are quite different with the kneading and the rub seen in Fig. 6b, c, respectively. Figure 6b shows the pressure from the front of the thumb finger is higher than the other parts of the finger during the kneading movement, while the pressure of the thumb finger is fairly uniform (about 68 kPa) during the rub movement as shown in Fig. 6c. The observed pressure distribution in the finger is somewhat similar with the previous reports in clinical observation [17, 20]. According to our measurements, the strain sensor based on flexible ferroelectric PVDF film prove to be sensitive for characterize the complex finger movement. It is expected to explore the skill of the human finger more precisely by using the proposed sensor, and it would also be helpful to develop the robot to replace human fingers in the future.

In conclusion, a 4 × 4 sensor array with 16 capacitor units based on the piezoelectric PVDF thin film has been fabricated and packaged with PDMS. The sensor array exhibits flexible and high sensitive properties. The hold-and-release output response of the sensor was obtained by applying impulse pressures with various frequencies, which indicated the sensor array could generate 20–300 mV voltage signals within 2 ms when applying a pressure in the range of 60–150 kPa. The obviously different pressure distributions in the finger during the finger movement of human hand have been observed by using the proposed sensor, which is expected to explore the skill of the human fingers more precisely.

## Abbreviations

PFM: Piezoresponse force microscopy
PVDF: Poly(vinylidene fluoride)
